# Association between the practice of fitness-related exercises and body image dissatisfaction in adolescents from Curitiba (PR), Brazil

**DOI:** 10.1590/1984-0462/2025/43/2023221

**Published:** 2024-09-06

**Authors:** Rinelly Pazinato Dutra, Yasmin Marques Castro, Maria Eduarda Santos de Almeida, Letícia Lamberty Pedrozo, João Venícios Tavares de Sousa, Murilo Bastos, Wagner de Campos, Michael Pereira da Silva

**Affiliations:** a Universidade Federal do Rio Grande, Rio Grande, RS, Brazil.; b Universidade Estadual do Centro-Oeste, Guarapuava, PR, Brazil.; c Universidade Federal do Paraná, Curitiba, PR, Brazil.

**Keywords:** Body image, Adolescent, Physical exercise, Body dissatisfaction, Physical fitness, Logistic models, Imagem corporal, Adolescente, Exercício físico, Insatisfação corporal, Aptidão física, Modelos logísticos

## Abstract

**Objective:**

The aim of this study was to analyze the association between participation in fitness-related exercises (FRE) and body image dissatisfaction (BID) in adolescents and evaluate the interaction between physical exercise and nutritional status in this association.

**Methods:**

A cross-sectional study was conducted in 2015 involving 799 adolescents (10–16 years old) from 14 public schools in Curitiba (PR), Brazil. BID was assessed using the Body Shape Questionnaire and the Silhouette Scale. The FRE was classified as “does not practice,” “practices ≤300 min/week,” and “practices >300 min/week” by the Physical Activity Questionnaire for Adolescents. Poisson and multinomial logistic regressions, adjusted for sex, sexual maturation, and nutritional status analyzed the association of FRE and BID.

**Results:**

The BID prevalence was 28.3%; 52.4% of the adolescents wanted to reduce their silhouettes; and 48.7% did not practice FRE. Adolescents who practiced FRE >300 min/week had a 28% higher prevalence for some level of BID (PR 1.28; 95%CI 1.08–1.52) and a 46% lower chance of wanting to reduce silhouettes (OR 0.54; 95%CI 0.35–0.82), compared to nonpractitioners. There was no interaction between FRE and nutritional status in association with BID.

**Conclusions:**

The adolescents who practice FRE >300 min/week are likely to have some level of BID and are less likely to report the desire to increase their silhouettes, regardless of their nutritional status.

## RESUMO

**Objetivo:**

Analisar a associação entre a participação em exercícios físicos relacionados ao *fitness* (EFRF) e a insatisfação com a imagem corporal (IIC) em adolescentes e avaliar a interação entre os exercícios físicos e o estado nutricional nesta associação.

**Métodos:**

Estudo transversal realizado em 2015 com 799 adolescentes (10 a 16 anos) de 14 escolas públicas de Curitiba (PR), Brasil. A IIC foi avaliada por meio do Body Shape Questionnaire e da Escala de Silhuetas. A participação em EFRF foi avaliada pelo Questionário de Atividade Física para Adolescentes e classificada em “não pratica”, “pratica ≤300 minutos/semana” e “pratica >300 minutos/semana”. As regressões de Poisson e logística multinomial, ajustadas por sexo, maturação sexual e estado nutricional, analisaram a associação entre EFRF e IIC.

**Resultados:**

A prevalência de IIC foi de 28,3%; 52,4% dos adolescentes queriam reduzir a silhueta e 48,7% não praticavam a EFRF. Adolescentes que praticavam EFRF >300 minutos/semana tiveram prevalência 28% maior para algum nível de IIC (razão de prevalência — RP 1,28; intervalo de confiança de 95% — IC95% 1,08–1,52) e chance 46% menor de querer reduzir silhuetas (OR 0,54; 95IC% 0,35–0,82), comparados aos não praticantes. Não houve interação entre os EFRF e o estado nutricional na associação com IIC.

**Conclusões:**

Os adolescentes que praticam EFRF >300 minutos/semana estão mais propensos a apresentar algum nível de IIC e têm menores chances de reportar o desejo de aumentar silhuetas, independentemente do estado nutricional.

## INTRODUCTION

Adolescence is widely recognized as a unique stage of human development, characterized by a series of biopsychosocial changes that significantly affect adult life.^1,2^ In the maturation process, there are several morphological changes that adolescents go through, such as the increase in adipose tissue, especially in girls, the increase in lean mass, especially in boys, bone growth, and consequent height increase in both sexes.^1,3^ Such transformations may considerably impact these adolescents’ body image perception.^4^

Body image is a multidimensional construct of how individuals perceive, describe, and feel about their physical appearance.^4,5^ Although the perception of body image is dynamic throughout life, adolescence is a susceptible period for its development since, together with biological transitions, new challenges arise related to accepting oneself, taking responsibility, and making choices and decisions.^5,6^ From this perspective, body image dissatisfaction (BID), which represents a desire to have a body different from the way it is perceived, is a known risk factor for psychological symptoms such as low self-esteem, anxiety, depression, and suicidal ideation, as well as for the occurrence of eating disorders.^4,7,8^

Several sociocultural, behavioral, and nutritional factors can influence BID.^3,8-10^ In the global context, systematic reviews estimate that between 32.2% and 83.0% of adolescents have some degree of BID and that dissatisfaction with body weight affects between 18.0% and 56.6% of adolescents.^11,12^ By associating BID with body weight in this age group, the prevalence of dissatisfaction due to being overweight or obese ranges from 44.0% to 83.0%, and low weight from 1.7% to 37.0%.^13^

In Brazil, studies that evaluated the perception of body image found BID prevalence ranging from 19.5% to 80.1%.^7,8,14-16^ BID is higher among girls and overweight or obese adolescents.^6,14-16^

Several studies have also explored the association between physical activity (PA) and adolescent body image perception.^5,17-19^ Although it is plausible to consider that PA might play an important role in promoting a more positive body image, most studies focus on general aspects of PA, such as the amount of time spent per week.^5,18^ In a prior study with the same population, no associations were identified between overall PA levels and BID, highlighting the necessity for further analyses exploring specific types of physical activities.^20^ Consequently, inconsistencies remain in the literature regarding the association between participation in specific types of PA and BID.

Some studies point out that different types of exercises, especially those with aesthetic purposes (fitness), may have some impact on BID; however, they still need to be investigated.^17,19^ Therefore, it is essential to understand the relationship between fitness-related exercises (FREs) and BID, considering the potential negative impact on adolescents’ lives, and provide relevant information and support strategies to promote health and well-being in the adolescent population.

Thus, this study aims to analyze the association between participation in FRE and BID in adolescents from Curitiba (PR). Additionally, we intended to evaluate the interaction between FRE and the nutritional status associated with BID.

## METHOD

This cross-sectional study uses data from the first evaluation of the project “Physical Activity and Health Risk Behaviors in Adolescents: A Prospective Cohort Study,” which began in 2015.

The sample included 799 adolescents of both sexes, aged between 10 and 16 years, enrolled in public schools in Curitiba (PR), Brazil. Sampling was stratified by clusters, considering the representativeness of each Curitiba Regional Education Area. Fourteen schools were drawn randomly within each educational region, and from each school, 4–6 classes from the sixth to the first year of high school were intentionally selected. All students in the chosen classes were invited to participate in the research.

The study was authorized by the State Department of Education of the State of Paraná SEED/PR and approved by the Ethics Committee for Research Involving Human Beings of the Federal University of Paraná (UFPR), under the Certificate of Ethical Presentation (CAEE: 39206214.3.0000.0102). The selected schools were previously contacted and received the necessary information about the objectives and procedures of the study. The acceptance of each school occurred through the signature and agreement of the director to carry out the research at the institution. Data collection took place between August and November 2015. Before participating, all participants obtained free and informed consent from their parents or legal guardians. Additionally, each participant also signed the Free and Informed Assent Term.

Adolescents answered questionnaires about sociodemographic information, PA and exercise participation, and perception of body image. Weight and height were measured, and the adolescents were invited to self-assess their sexual maturation. All data collection procedures took place in the school environment and were conducted by a trained team of members and collaborators of the Center for Studies in Physical Activity and Health at UFPR.

Adolescents were categorized into three groups, considering the age groups:

a) 10–11 years,
b) 12–13 years, and
c) 14–16 years.

The socioeconomic status was evaluated using the questionnaire of the Brazilian Association of Research Companies (ABEP), which has been divided into A, B1, B2, C1, C2, D, and E classes. To facilitate the analytical process, the socioeconomic status was aggregated and categorized into high (A), middle (B1, B2, C1, and C2), and low (D and E). The participants’ sexual maturation was assessed using the method of self-assessment of pubic hair development, using drawings as a reference and classifying them into five stages.^21,22^

The nutritional status of the participants was assessed using the calculation of the body mass index (BMI). The classification followed the categories based on the BMI z-scores proposed by the World Health Organization, where z-score <-2 SD=low weight, between ≥-2 SD and ≥+1=normal weight, and >+1 SD overweight/obese. Considering that only two adolescents were underweight, and for analytical purposes, we used only the normal weight and overweight/obesity categories.

Body Shape Questionnaire (BSQ) evaluated the BID, which was validated and adapted for the population of Brazilian adolescents.^23^ The BSQ consists of 34 questions that assess the perception of dissatisfaction with body appearance over the last 4 weeks. Each question offers a scale of responses ranging from “never” (1) to “always” (6). The dissatisfaction score is calculated by adding the response values, resulting in a score ranging from 34 to 204. Based on this score, adolescents were classified into two categories: “Satisfied” (score <80) and “With some level of dissatisfaction” (score ≥80).

In addition to using the BSQ, the Silhouette Scale, validated for Brazilian adolescents, was used as an additional means to identify dissatisfaction with body appearance.^24^ The instrument consists of two questions that address the perception of the current body appearance (current silhouette score) and the desired appearance (ideal silhouette score). For each question, nine silhouettes were presented, representing a variety of body conditions, from extreme thinness to obesity levels, encompassing both sexes. The instructions given to the adolescents consisted of selecting the silhouette that best corresponded to their current body appearance and, subsequently, the silhouette that best represented the desired (ideal) appearance. The BID assessment was obtained by calculating the difference between the current and ideal silhouette scores. Adolescents who presented values equal to zero were classified as “Satisfied.” Those with positive values were classified in the “Want to decrease” category, while those with negative values were categorized as “Want to increase.”

The Physical Activity Questionnaire for Adolescents (QAFA) assessed participation in FREs.^25^ This instrument has a list of activities and a space to add others not listed. The adolescent was asked to answer how many days per week and how long per day they practiced these activities in the previous week. For analysis purposes, they were grouped as FREs: bodybuilding, gymnastics, sit-ups, running, and walking as exercise. The variable was then categorized into “does not practice,” “practices up to 300 min/week,” and “practices more than 300 min/week.”

Statistical analysis of the data was first performed descriptively, presenting the characteristics of the investigated sample in terms of absolute and relative frequency. The prevalence of the BID outcome (through the BSQ and Silhouette Scale) and the frequency of participation in FRE were also calculated. The Poisson regression with robust error control by clusters (school) verified the association between FRE and the BID by BSQ, with the result expressed in prevalence ratios (PR). Multinomial logistic regression verified the association between FRE and BID by the Silhouette Scale, with results in odds ratio (OR). Both analyses were adjusted for sex, sexual maturation, and nutritional status. In addition, we tested the interaction between FRE and nutritional status in association with the BSQ body dissatisfaction score. The model was adjusted for sex and sexual maturation to control possible confounding effects. Confidence intervals of 95% (95% CI) were presented for all analyses, and p < 0.05 was considered statistically significant. The entire analytical process was conducted using the Stata MP 16 software.

## RESULTS

The study sample consisted of 799 adolescents, with 51.3% (n=410) being female. The predominant age group was between 14 and 16 (47.3%) years, and most adolescents were of middle socioeconomic status (67.2%), 58.4% were classified as normal weight, and 36.2% were in the fourth maturational stage (Table 1).

**Table 1:** Characterization of the sample of adolescents enrolled in public schools in Curitiba (PR), Brazil (n=799).

Variables	N	%
Sex		
Male	389	48.7
Female	410	51.3
Age group (years)		
10–11	203	25.4
12–13	218	27.3
14–16	378	47.3
Socioeconomic level*		
High	55	6.9
Medium	536	67.2
Low	207	25.9
BMI		
Normal weight	467	58.4
Overweight/obesity	332	41.5
Sexual maturation*		
Stage 1	18	2.4
Stage 2	115	15.3
Stage 3	244	32.4
Stage 4	273	36.2
Stage 5	103	13.7

BMI: body mass index.

*Variables with missing data.

**Figure 1:**
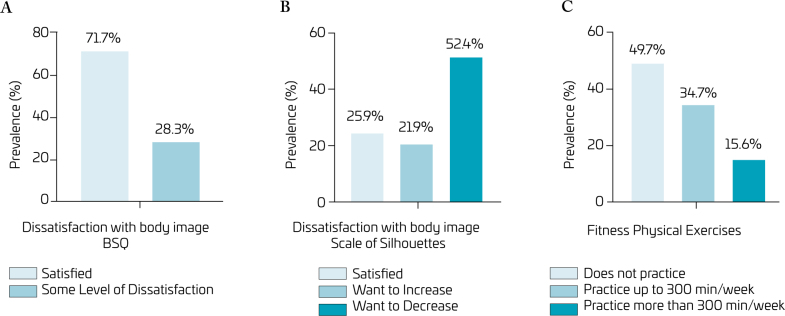
Prevalence of body dissatisfaction according to the Body Shape Questionnaire (BSQ) and the Silhouette Scale, and prevalence of participation in fitness-related exercises.

Figure 1 shows the prevalence of BID outcomes according to the BSQ (A) and the Silhouette Scale (B), as well as participation in FRE (C). It was observed that 28.3% of the adolescents had some level of BID, and more than half wanted to decrease their silhouette (52.4%). Regarding FRE, 34.7% practiced it up to 300 min/week, and 15.6% practiced it for more than 300 min/week.

When analyzing the association between participation in FREs and BID, according to the BSQ, it was seen that adolescents who practiced more than 300 min/week had a 28% higher prevalence of some level of BID (PR 1.28; 95%CI 1.08–1.52) when compared to those who did not exercise (Table 2). Regarding the Silhouette Scale, those who reported exercising >300 min/week had a 46% lower chance of being in the group that reported the desire to increase it compared to their peers who did not exercise (OR 0.54; 95%CI 0.35–0.82) (Table 3).

**Table 2:** Association between body dissatisfaction by Body Shape Questionnaire and participation in fitness-related exercises.

Variable	Crude analysis		Adjusted analysis*	

	PR (95%CI)	p-value	PR (95%CI)	p-value
Fitness physical exercise				
Does not practice	1		1	
Practice up to 300 min/week	0.99 (0.78–1.25)	0.930	0.99 (0.81–1.21)	0.921
Practice >300 min/week	1.36 (1.15–1.61)	<0.001	1.28 (1.08–1.52)	0.004

PR: prevalence ratio; CI: confidence interval.

*Adjusted for sex, sexual maturation, and nutritional status.

**Table 3:** Association between body dissatisfaction according to the Silhouette Scale and participation in fitness-related exercises.

Variable	Want to increase				Want to decrease			

	Crude		Adjusted*		Crude		Adjusted*	

	OR (95%CI)	p-value	OR (95%CI)	p-value	OR (95%CI)	p-value	OR (95%CI)	p-value
Fitness physical exercise								
Does not practice	1		1		1		1	
Practice up to 300 min/week	1.08 (0.73–1.61)	0.698	1.02 (0.68–1.52)	0.936	1.25 (0.77–2.02)	0.361	1.19 (0.70–2.03)	0.518
Practice >300 min/week	0.59 (0.40–0.88)	0.010	0.54 (0.35–0.82)	0.004	1.01 (0.57–1.80)	0.953	0.90 (0.47–1.75)	0.766

OR: *Odds Ratio*; CI: confidence interval.

*Adjusted for sex, sexual maturation, and nutritional status.

The analysis of the interaction between FRE and nutritional status associated with the BID measured by BSQ was insignificant. For overweight/obese adolescents who exercised for up to 300 min/week, a PR 1.08 (95%CI 0.44–2.63, p=0.872) was obtained, whereas, for those with overweight/obesity and who exercised more than 300 min/week, the PR was 0.74 (95%CI 0.42–1.30, p=0.30).

## DISCUSSION

The present study analyzed the association between FRE participation and adolescent BID. The results revealed that about three out of ten adolescents reported body dissatisfaction. Regarding the Silhouette Scale, two out of ten participants wanted to increase it, while more than half of the sample wanted to decrease it. Half of the students were involved in some of the listed FRE activities, with approximately 34.7% spending up to 300 min and 15.6% more than 300 min/week. The analysis also revealed that adolescents who practiced more than 300 min/week had a higher prevalence of some level of BID and lower chances of being in the group that reported the desire to increase their silhouette.

Our results aligned with similar studies about the prevalence of BID in adolescents. In Brazil, the BID varied between 17% and approximately 75% of adolescents.^7,10,14^ A similar situation can be observed in international surveys: from 26.4% in a study in Eastern Europe to 65.9% in Spain.^26,27^ Although the Brazilian regions and the countries studied present very different economic and sociocultural aspects, dissatisfaction with the image perceived by young people is a point of convergence. The social pressure exerted by family members, friends/colleagues, other entities in society in general, and the media, especially on social networks, set up powerful mechanisms for internalizing idealized beauty standards, stimulating social comparisons, and decreasing the appreciation of one’s own body.^4,9^

Although about half of the adolescents reported not performing any FRE, it is essential to emphasize that these results show a relatively optimistic scenario compared to the global situation. Despite the recommendation of the World Health Organization of 60 min of PA daily, only 7.5% of Brazilian adolescents are physically active, and four out of five are insufficiently active worldwide.^28,29^ Regarding the prevalence of FREs, studies that address this analysis in adolescents are less frequent since most articles evaluated the total time spent in PA, without describing the type of PA practiced.^10,28,29^ However, in a study conducted with 637 participants, the prevalence of FRE was around 14%.^17^ Studies from a systematic review showed that the main barriers for these adolescents to be physically active are the negative influence of the social and family nucleus, the BID, concerns about exposure, social norms, low physical fitness, and fatigue.^30^

The PA is widely recognized as a powerful mechanism for promoting health and quality of life for adolescents, and is encouraged by health agencies as a way to promote a healthy lifestyle and improve physical and mental well-being in this age group.^25,28-30^ The existing body of evidence also shows that physically active adolescents tend to have lower BID scores.^5,18,27^ This association seems to occur due to multifactorial issues, such as improvement in physical fitness, reduction in body weight, improvement in self-esteem, confidence, perception, and acceptance of one’s own body.^5,18,27^ On the contrary, there are gaps regarding the direction of these associations since body dissatisfaction can also predict PA participation.^5,17^

At the same time, the search for exercises with aesthetic purposes adds an intriguing layer to this complex relationship. Some studies have shown different perspectives, suggesting that the type and the time spent in the activity may be relevant factors that increase BID.^5,17,19^ Specifically, involvement in fitness exercises significantly impacts the perception of body image, as its performance may be linked to the search for the ideal body imposed by social standards.^19,31^

The trend known as “fitspiration” is a predominant feature in the social media scene to motivate users to adopt a healthy lifestyle through diet and exercise, aiming to achieve a positive perception of body image.^31^ This movement demonstrates a great potential for influencing physical and mental health; however, what may seem edifying is often related to the objectification of bodies and the idealization of unattainable beauty standards. These norms negatively affect the perception of body image and may lead to mental health issues and eating disorders.^8,31^

In this context, some investigations have pointed out that adolescents involved in PA for aesthetic and weight loss purposes demonstrated higher rates of body dissatisfaction and an increased desire to achieve a slimmer silhouette compared to those who participated in activities not related to aesthetic purposes or who were physically inactive.^8,17^ These findings also suggest that this relationship is regulated by several mechanisms, including characteristics, types, and motivations underlying the practice of these activities.^17^

Our results contribute significantly to this debate, showing that adolescents who spend more than 300 min/week in FREs are likely to report some BID level. In addition, no evidence of interaction was found between participation in FREs and nutritional status in association with BID in adolescents. Although the literature is consistent about the associations between body weight and body dissatisfaction, especially for obese adolescents, the observed effect of fitness exercises on BID seems to have occurred regardless of BMI classification.^5^ These aspects reinforce that the time and type of PA practiced are important predictors of body dissatisfaction in this population, corroborating previous findings.^17,19^

It is essential to interpret the results of this study considering its limitations and strengths. First, although the selection of schools was random, the intentional choice of the classes may reduce the sample’s representativeness. However, it is essential to point out that sample weights were used to mitigate issues of representativeness. Moreover, including students from different social classes and neighborhoods of Curitiba (PR), Brazil, allowed for greater sample diversity. Furthermore, using self-reported questionnaires to assess adolescent behavior can affect the accuracy of the measured aspects. For example, the instrument to evaluate exercise participation may have overestimated this variable.

For the BID evaluation, we opted for the joint use of the instruments because they approach the perception of body image from different and complementary perspectives. While the BSQ comprehensively assesses behavioral and psychological indicators associated with body dissatisfaction, the Silhouette Scale specifically addresses aesthetic factors related to body shape dissatisfaction.^23,24^ Thus, combining both instruments allows a more complete and accurate assessment of body image perception.

Furthermore, it is important to contextualize our findings in the broader context of health. Although our study discussed FREs practiced for aesthetic purposes, these activities also play a crucial role in promoting physical fitness and health, depending on other variables such as frequency, intensity, time, and prescribed activity.^32^ However, this study did not evaluate the practitioner’s intentionality and specific prescription characteristics of these FREs. Future investigations can explore these additional factors to understand better the relationship between physical exercise and adolescent BID.

Finally, it is necessary to interpret the findings cautiously since the cross-sectional design does not allow for establishing a cause-and-effect relationship between participation in FREs and BID, particularly considering the temporal aspect, a crucial criterion for causal inference.^33^ It is possible that BID influences adherence to exercises or that frequent participation in FREs may affect the perception of body image. To better understand these aspects, future investigations should adopt longitudinal designs or mixed methods that allow a deeper investigation of the FRE and BID association.

This study showed that adolescents who practice more than 300 min/week of FREs are likely to report some level of BID and are less likely to report the desire to increase their silhouette, regardless of their nutritional status. This study provides important insights into the relationship between the amount and type of exercise and adolescent body image perception. Such findings are relevant for this population’s physical and mental health since BID is associated with several psychological and physical problems. These results may provide subsidies for health promotion interventions and policies aimed at adolescents, considering the need for specific approaches for those intensely engaged in FREs.

## Data Availability

The database that originated the article is available with the corresponding author.

## References

[1] Norris SA, Frongillo EA, Black MM, Dong Y, Fall C, Lampl M (2022). Nutrition in adolescent growth and development. Lancet.

[2] Sawyer SM, Azzopardi PS, Wickremarathne D, Patton GC (2018). The age of adolescence. Lancet Child Adolesc Health.

[3] Kobylińska M, Antosik K, Decyk A, Kurowska K, Skiba D (2022). Body composition and anthropometric indicators in children and adolescents 6–15 years old. Int J Environ Res Public Health.

[4] Dion J, Blackburn ME, Auclair J, Laberge L, Veillette S, Gradeault M (2015). Development and aetiology of body dissatisfaction in adolescent boys and girls. Int J Adolesc Youth.

[5] Gualdi E, Rinaldo N, Zaccagni L (2022). Physical activity and body image perception in adolescents: a systematic review. Int J Environ Res Public Health.

[6] Fortes LS, Conti MA, Almeida SS, Ferreira ME (2013). Insatisfação corporal em adolescentes: uma investigação longitudinal. Rev Psiq Clin.

[7] Claumann GS, Pinto AA, Silva DA, Pelegrini A (2018). Prevalência de pensamentos e comportamentos suicidas e associação com a insatisfação corporal em adolescentes. J Bras Psiquiatr.

[8] Leal GV, Philippi ST, Alvarenga MS (2020). Unhealthy weight control behaviors, disordered eating, and body image dissatisfaction in adolescents from São Paulo, Brazil. Braz J Psychiatry.

[9] Scully M, Swords L, Nixon E (2023). Social comparisons on social media: online appearance-related activity and body dissatisfaction in adolescent girls. Ir J Psychol Med.

[10] Iepsen AM, Silva MC (2014). Prevalência e fatores associados à insatisfação com a imagem corporal de adolescentes de escolas do Ensino Médio da zona rural da região sul do Rio Grande do Sul, 2012. Epidemiol Serv Saúde.

[11] Côrtes MG, Meireles AL, Friche AA, Caiaffa WT, Xavier CC (2013). O uso de escalas de silhuetas na avaliação da satisfação corporal de adolescentes: revisão sistemática da literatura. Cad Saude Publica.

[12] San Martini MC, Assumpção D, Barros MB, Mattei J, Barros AA (2022). Prevalence of body weight dissatisfaction among adolescents: a systematic review. Rev Paul Pediatr.

[13] Jiménez Flores P, Jiménez Cruz A, Bacardi Gascón M (2017). Body-image dissatisfaction in children and adolescents: a systematic review. Nutr Hosp.

[14] Carvalho GX, Nunes AP, Moraes CL, Veiga GV (2020). Insatisfação com a imagem corporal e fatores associados em adolescentes. Cien Saude Colet.

[15] Moehlecke M, Blume CA, Cureau FV, Kieling C, Schaan BD (2020). Self-perceived body image, dissatisfaction with body weight and nutritional status of Brazilian adolescents: a nationwide study. J Pediatr (Rio J).

[16] Rocha RP, Galvão PP, der Meer Sanchez ZM, Rebouças LN, Castro AR, Santos LE (2022). Body dissatisfaction, drug use, and associated factors among adolescents in three Brazilian cities. Rev Lat Am Enfermagem.

[17] Fernández-Bustos JG, Infantes-Paniagua A, Gonzalez-Martí I, Contreras-Jordán OR (2019). Body dissatisfaction in adolescents: differences by sex, bmi and type and organisation of physical activity. Int J Environ Res Public Health.

[18] Laudańska-Krzemińska I, Krzysztoszek J, Naczk M, Gajewska E (2020). Physical activity, physical fitness and the sense of coherence-their role in body acceptance among Polish adolescents. Int J Environ Res Public Health.

[19] Laus MF, Costa TM, Almeida SS (2013). Body image dissatisfaction and aesthetic exercise in adolescents: are they related?. Estud Psicol (Natal).

[20] Fantineli ER, Silva MP, Campos JG, Malta NA, Pacífico AB, Campos W (2020). Imagem corporal em adolescentes: associação com estado nutricional e atividade física. Ciênc Saúde Colet.

[21] Bojikian LP, Massa M, Martin RH, Teixeiral CP, Kiss MA, Bohmel MT (2002). Auto-avaliação puberal feminina por meio de desenhos e fotos. RBAFS.

[22] Martin RH, Uezu R, Parra SA, Arena SS, Bojikian LP, Böhme MT (2001). Auto-avaliação da maturação sexual masculine por meio da utilização de desenhos e fotos. Rev Paul Educ Fis.

[23] Conti MA, Cordás TA, Latorre MR (2009). A study of the validity and reliability of the Brazilian version of the Body Shape Questionnaire (BSQ) among adolescents. Rev Bras Saúde Mater Infant.

[24] Conti MA, Latorre MR (2009). Estudo de validação e reprodutibilidade de uma escala de silhueta para adolescentes. Psicol Estud.

[25] Farias JC, Lopes AS, Mota J, Santos MP, Ribeiro JC, Hallal PC (2012). Validade e reprodutibilidade de um questionário para medida de atividade física em adolescentes: uma adaptação do Self-Administered Physical Activity Checklist. Rev Bras Epidemiol.

[26] Karchynskaya V, Kopcakova J, Madarasova GA, Klein D, Winter AF, Reijneveld SA (2022). Body image, body composition and environment: do they affect adolescents’ physical activity?. Eur J Public Health.

[27] Añez E, Fornieles A, Fauquet-Ars J, López-Guimerà G, Puntí-Vidal J, Sánchez-Carracedo D (2018). Body image dissatisfaction, physical activity and screen-time in Spanish adolescents.. J Health Psychol.

[28] Araujo RH, Werneck AO, Barboza LL, Ramírez R, Martins CM, Tassitano RM (2022). Prevalence and sociodemographic correlates of physical activity and sitting time among south American adolescents: a harmonized analysis of nationally representative cross-sectional surveys. Int J Behav Nutr Phys Act.

[29] Guthold R, Stevens GA, Riley LM, Bull FC (2020). Global trends in insufficient physical activity among adolescents: a pooled analysis of 298 population-based surveys with 1·6 million participants. Lancet Child Adolesc Health.

[30] Martins J, Costa J, Sarmento H, Marques A, Farias C, Onofre M (2021). Adolescents’ perspectives on the barriers and facilitators of physical activity: an updated systematic review of qualitative studies. Int J Environ Res Public Health.

[31] Tiggemann M, Zaccardo M (2015). “Exercise to be fit, not skinny”: the effect of fitspiration imagery on women’s body image. Body Image.

[32] American College of Sports Medicine (2014). ACSM’s guidelines for exercise testing and prescription.

[33] Hall W (2024). Austin bradford hill’s ‘environment and disease: association or causation’. Addiction.

